# Sorption behavior of strontium and europium ions from aqueous solutions using fabricated inorganic sorbent based on talc

**DOI:** 10.1038/s41598-024-69824-3

**Published:** 2024-08-26

**Authors:** M. R. Abass, R. A. Abou-Lilah, L. M. S. Hussein

**Affiliations:** Hot Laboratories, and Waste Management Centre, Egyptian Atomic Energy Authority, Cairo, 13759 Egypt

**Keywords:** Sr(II), Eu(III), SnMoT sorbent, Reaction kinetics, Isotherm, Desorption, Environmental sciences, Environmental social sciences

## Abstract

Sorption of Sr(II) and Eu(III) from aqueous solutions was studied using tin molybdate talc sorbent synthesized by the precipitation technique. The synthesized sorbent was characterized using different analytical tools, such as; FT-IR, SEM, XRD, XRF, TGA, and DTA. The sorption studies applied to Sr(II) and Eu(III) include the effects of shaking time, pH, concentrations, and saturation capacity. The sorption of Sr(II) and Eu(III) depends on pH, reaction kinetics obey the pseudo-2nd-order model, and the Langmuir model is better suited for the sorption isotherm. The thermodynamic parameters reflect an endothermic and spontaneous sorption process. Desorption studies showed that 0.1 M HCl was the best desorbing agent for the complete recovery of Sr(II) (96.8%) and Eu(III) (92.9%). Finally, the obtained data illustrates that the synthesized sorbent can be applied and used as an efficient sorbent for the sorption of Sr(II) and Eu(III) from aqueous solutions and can be used as a promising sorbent to remove Sr(II) and Eu(III).

## Introduction

Various processes in power plants, recycling facilities, research centers, and using radioisotopes in industry and diagnostic medicine produce radwaste^1^. The development and use of nuclear energy are accompanied by the generation of significant amounts of radioactive waste that cannot be ignored^2^. These streams may also contain other harmful and poisonous substances, such as heavy metals, organic material from decontamination processes, and radioisotopes. Radioactive nuclides that were emitted found their way into the soil, homes, trees, plants, water, and other buildings^4–8^. The radioisotope concentrations in these wastes must be reduced to acceptable levels before being released into the environment^1^.

Owing to its high fission yield (4.5%), medium half-life (28.9 y), emission of high-intensity beta rays, and high water solubility, radioactive strontium (^90^Sr) is one of the most dangerous radionuclides^9^. In addition, ^90^Sr readily accumulates in human bones through the food chain and has a chemistry similar to calcium. This can result in blood and bone malignancies^10,11^. The most crucial step in achieving the upper radioactivity limit set by the Environmental Protection Agency for ^90^Sr in drinking water is removing very low concentrations of the metal from natural water that contains high concentrations of competing cations (Ca^2+^ and Mg^2+^). This limit is 0.3 Bq/L, or roughly 0.057 ppq (parts per quadrillion)^12^. ^152^Eu (t_1/2_ = 13.54 y) and ^154^Eu (t_1/2_ = 8.67 y) are mostly created as fission products from radioactive waste. At the same time, they can be made by neutron irradiating isotopically enriched ^152^Sm_2_O_3_ and neutron activating nuclear reactor control rods, respectively^13^. Europium is used in several medical diagnoses to detect renal and cardiac diseases and as a test to control blood hormone levels^14^.

Sr(II) and Eu(III) have been removed from the contaminated water using various techniques including adsorption^15^, chemical precipitation^16–18^, solvent extraction^19^, membrane processes^20^, and ion exchange^21–23^, etc. Among these techniques for treating radioactive wastewater, considering its ease of handling, cheap cost, high efficiency, and high treatment capacity, adsorption may hold greater promise than many other approaches for converting trash into a more stable solid form with less volume. Adsorption is one of the main methods for eliminating radionuclides with great availability and economic viability. Consequently, creating numerous stable adsorbents is essential for decontaminating Eu(III) and Sr(II), particularly inorganic ones with strong thermal, chemical, and radiation stability, high adsorption affinity and efficiency, and good selectivity^24^. Strontium and europium have been removed from aquatic environments using a variety of organic and inorganic adsorbents, including natural zeolites, porous carbon composites, graphene oxides, ammonium molybdophosphate composites, hydroxyapatites, and sodium titanates^25–27^.

Talc is branded by its 2:1 sheet structure, consisting of 2 layers of the Si–O tetrahedron and a layer of Mg(OH)_2_^28^. The talc (T) block is broken into T-powder, then milled and separated. It is extensively utilized in sectors including paper, plastic, rubber, food, medicine, cosmetics, and ceramics^29^. Talc can also be utilized as an adsorbent to remediate radioactive wastewater due to its high porosity, large specific surface area, rod-like shape, and abundant hydrophilic Si–OH^30–32^.

Talc composites modified with other elements such as modified talcum^33^, talc phosphogypsum ferri-silicate sorbent^30^, Fe_3_O_4_/Talc^34^, P(AA-AN)-talc^28^, and iron phosphate talc (IPT)^35^. The novelty of this study includes the impregnation of tin and molybdate groups inside talc layers for the high-efficiency sorption of Sr(II) and Eu(III) from aqueous solutions that have not yet been previously studied by scholars. In this study, SnMoT sorbent was synthesized using the precipitation technique and used to remove Sr(II) and Eu(III) from aqueous solutions by batch methods^36,37^.

This work aimed to synthesize tin molybdate talc (SnMoT) sorbent. The synthesized sorbent was characterized by utilizing various instruments. The possible uses of this sorbent in solid-phase extraction of Sr(II) and Eu(III) under various batch experiment conditions were assessed using their aqueous solutions.

## Experimental

### Materials and instruments

The main reagents synthesizing SnMoT sorbent were SnCl_2_·2H_2_O and Na_2_MoO_4_·2H_2_O, obtained from Sigma-Aldrich and Loba Chemie (India), respectively. SrCl_2_ (Merck, Germany), Eu_2_O_3_, HNO_3_, and HCl (Merck, Germany), as well as NaOH and NH_3_ (El-Nasr Co, Egypt). Na_5_P_3_O_10_ (Goway, China). Both chemicals and components used in this article possess analytical grades devoid of additional purification. For all experimentations, composites, and solutions were prepared using demineralized water. Bruker D2 Phaser II, Germany, and Alpha II Bruker, Germany, were used to evaluate SnMoT sorbent using X-ray diffraction (XRD) and Fourier-transform infrared spectroscopy (FT-IR), respectively. Differential thermal analysis (DTA) and thermogravimetric analysis (TGA) of the SnMoT sorbent were conducted with a Shimadzu DTG-60H instrument. For the TGA/DTA analysis, 20 mg samples were heated from room temperature up to 700 °C at a rate of 10 °C min^−1^ under a nitrogen atmosphere using alumina powder as the reference. The elemental analysis of SnMoT sorbent was detected using Philips sequential X-ray spectrometer-2400. The % of SiO_2_, MgO, MoO_3_, SnO_2_, Fe_2_O_3_, and Al_2_O_3_ was calculated based on the quantitative application procedure for Super-Q. The morphology of SnMoT sorbent was determined using the scanning electron microscopy (SEM) model Philips XL 30.

### Preparation

#### Preparation of reagents

A talc-dispersed solution was prepared by dissolving 30 g talc powder in 300 mL DDW for 1 h in the presence of 0.3 g Na_5_P_3_O_10_ as a dispersing agent. Na_2_MoO_4_·2H_2_O solution (0.3 M) was prepared by dissolving 14.51 g Na_2_MoO_4_·2H_2_O powder in 200 mL DDW for 1 h. SnCl_2_·2H_2_O solution (0.3 M) was prepared by dissolving 13.54 g SnCl_2_·2H_2_O powder in 200 mL HCl (4 M) for 2 h.

#### Preparation of SnMo sorbent

The preparation of the SnMo sorbent was done using a co-precipitation technique. In this method, SnCl_2_·2H_2_O solution (0.3 M) dropwise to Na_2_MoO_4_·2H_2_O solution (0.3 M) by a volumetric ratio equal unity at constant stirring. After complete addition, a brownish-red color was obtained. Ammonia solution (10% v/v) was dropwise to a mixed solution until a precipitate formed at pH (7.2), and the reaction mixture was diluted to one litter and allowed to settle through one day. The residue was washed several times to remove free chloride ions. The residue was dried for 24 h at 55 ± 1 °C, sieved for different mesh sizes, and then stored at 25 ± 1 °C.

#### Preparation of talc (T) sorbent

A talc-dispersed solution was precipitated using (10% v/v) ammonia solution dropwise. The residue was dried for 24 h at 55 ± 1 °C, sieved for different mesh sizes, and then stored at 25 ± 1 °C.

#### Preparation of SnMoT sorbent

The SnMoT sorbent was prepared as follows: Na_2_MoO_4_·2H_2_O solution (0.3 M) and SnCl_2_·2H_2_O solution (0.3 M) were added dropwise to the talc-dispersed solution by volumetric ratio Sn:Mo:T equal to 1:1:2 at constant stirring for 2 h. SnMoT was precipitated by adding (10% v/v) ammonia solution dropwise. The residue was washed, dried at 55 ± 1 °C for 24 h, grained, sieved for various mesh sizes, and stored at 25 ± 1 °C.

### The best adsorbent selection

To select the best sorbent for % sorption, the % sorption of Sr(II) and Eu(III) onto produced sorbents by various volumetric ratios was carried out by shaking 0.1 g solid with 10 mL of Sr(II) and Eu(III) (100 mg/L) and V/m = 100 mL/g at 25 ± 1 °C for 24 h. After this time, the shaker is turned off, and the solution and solid are immediately separated. The initial and final concentrations (C_o_ and C_f_) of Sr(II) and Eu(III) used were measured using an atomic absorption spectrophotometer (Buck Scientific, VGP 210) and Shimadzu UV–visible Recording Spectrophotometer (UV-160A) manufactured and supplied by Shimadzu Kyoto, Japan. The % sorption can be calculated by using (Eq. 1)^38,39^:

1 $$\% {\text{sorption }} = \left( {\frac{{{\text{C}}_{{\text{o}}} - C_{{\text{f}}} }}{{{\text{C}}_{{\text{o}}} }}} \right)100$$

The results in Table 1 indicate the sequence order for the sorption of Sr(II) and Eu(III) sorption onto different prepared sorbents: Sr(II) ˃ Eu(III). Also, sorbent no. 3 (SnMoT) is the best sorbent and is used for all experimental work.

**Table 1:** Conditions for the synthesis of different sorbents and their % sorption of Sr(II) and Eu(III) (100 mg/L, V/m = 100 mL/g, and shaking time 24 h) at room temperatures.

Samples	SnCl_2_·2H_2_O (0.3 M)	Na_2_MoO_4_·2H_2_O (0.3 M)	Talc (10% w/v)	% sorption	
				Sr(II)	Eu(III)
S-1	0	0	200 mL	65.8	40.8
S-2	100 mL	100 mL	0	78.9	60.5
S-3	100 mL	100 mL	200 mL	99.2	76.6

### Chemical stability

To test the sorbent's stability to various solvents, 50 mg of SnMoT was shaken with 50 mL of H_2_O, HNO_3_, HCl, and NaOH at varying concentrations [1–4 M]. Shake the appropriate solution periodically for about a week at 25 ± 1 °C. Infrared lamps were used to dry the filtrates before being examined gravimetrically^37,40,41^.

### Sorption studies

Many parameters like pH (1–8), concentration (50–1000 mg/L), agitating time (2–270 min), and temperature (25–65 °C) were checked to determine the ideal state for sorption. Batchwise contact was made between the sorbent and the sorbate solution; the samples were filtered out of the solution following sorption. All equilibrium measurements were carried out by shaking 0.1 g of SnMoT sorbent with 10 mL of Sr(II) and Eu(III) of the initial concentration of 100 mg/L with V/m = 100 mL/g in an agitator thermostat (Kottermann D-1362, Germany). The average of two duplicate experiments constituted all of the provided experimental results in this inquiry. The adsorption capacities at equilibrium (q_e_, mg/g) of Sr(II) and Eu(III) retained on the SnMoT sorbent were determined utilizing the next equation, respectively^42–44^:

2 $${\text{q}}_{{\text{e}}} = {\text{(C}}_{{\text{o}}} - {\text{C}}_{{\text{e}}} )\frac{{\text{V}}}{{\text{m}}}$$

where C_o_ and C_e_ are the initial and equilibrium concentrations of Sr(II) and Eu(III) in the aqueous solution (mg/L); V is the volume of the solution (L), and m is the mass of the dried adsorbent (g).

### Kinetic analysis

To elucidate the workings of the adsorption process, the pseudo-1st-order (PFO) (Eq. 3) and pseudo-2nd-order (PSO) (Eq. 4). The PFO model, which represents a solid–liquid system, is based on the adsorbent's capacity for adsorption^45^. The solid-phase adsorption capacity and the number of active centers on the adsorbent surface serve as the PSO model's foundation^46^.

3 $${\text{q}}_{{\text{t}}} = {\text{q}}_{{\text{e}}} \left[ {1 - \exp ( - {\text{K}}_{{\text{1}}} {\text{t}})} \right]$$

4 $${\text{q}}_{t} = \frac{{{\text{q}}_{e}^{2} {\text{K}}_{2} t}}{{1 + {\text{q}}_{e} {\text{K}}_{2} t}}$$

t: time (min), K_1_ and K_2_: the rate constants of the PFO (min^−1^) and PSO model (g/mg_._min), respectively. Initial rates for the PFO and PSO adsorption models were computed utilizing Eqs. (5) and (6), respectively.

5 $${\text{H}}_{1} = {\text{K}}_{1} {\text{q}}_{e}$$

6 $${\text{H}}_{2} = {\text{K}}_{2} {\text{q}}_{e}^{2}$$

H_1_ and H_2_: the initial PFO and PSO adsorption rates (mg/g.min), respectively.

### Isotherm modeling

The concentration data obtained to acquire the isotherms of the Sr(II) and Eu(III) loaded onto SnMoT sorbent were examined using nonlinear versions of the Langmuir (Eqs. 7 and 8) and Freundlich (Eq. 9) models. Sorption isotherm measurements were made in the presence of initial concentrations (50–1000 mg/L) and pH 6 and 4 for Sr(II) and Eu(III), respectively. The Langmuir model postulates that the adsorbent surface's active adsorption centers are uniformly distributed^47^. The Freundlich model explains adsorbent surface heterogeneity, which also offers information on hyperbolic adsorption behavior^48^.

7 $${\text{q}}_{{\text{e}}} = {\text{q}}_{{\text{m}}} {\mkern 1mu} \frac{{{\text{K}}_{{\text{L}}} {\text{C}}_{{\text{e}}} }}{{1 + {\text{K}}_{{\text{L}}} {\text{C}}_{{\text{e}}} }}$$

8 $${\text{R}}_{{\text{L}}} = \frac{1}{{1 + {\text{K}}_{{\text{L}}} {\text{C}}_{{\text{o}}} }}$$

9 $${\text{q}}_{{\text{e}}} = {\text{K}}_{{\text{F}}} {\text{C}}_{{\text{e}}}^{{\frac{{\text{1}}}{{\text{n}}}}}$$

The maximum adsorption capacity (mg/g), Langmuir isotherm parameter, and the separation factor are denoted by q_m_, K_L_, and R_L_, respectively. K_F_ and 1/n are Freundlich constant and adsorbent surface heterogeneity, respectively.

To estimate the degree of difference (χ^2^) between the experimental data and the calculated data chi-square analysis was applied, which is calculated by the following equation^49^.

10 $$\upchi ^{2} = \sum\limits_{{{\text{i}} = 1}}^{{\text{n}}} {\frac{{\left( {{\text{q}}_{{{\text{exp}}.}} - {\text{q}}_{{{\text{cal}}.}} } \right)^{2} }}{{{\text{q}}_{{{\text{cal}}.}} }}}$$

where q_cal._ and q_exp._ (mg/g) are the amount of ion adsorbed and the experimental equilibrium uptake amount, respectively. A smaller χ^2^ value indicates a better-fitting isotherm.

### Effect of temperature

Calculating thermodynamic parameters can help determine whether or not the adsorption process is spontaneous. Furthermore, using thermodynamic parameters at different reaction temperatures (298, 313, and 338 K), we can easily show the temperature effect on the Sr(II) and Eu(III) sorbed onto SnMoT sorbent. The experiment was conducted at the initial concentration of studied cations, 200 mg/L, pH 6 and 4 for Sr(II) and Eu(III), respectively, and shaking time = 210 min. For the calculating of ∆H° (enthalpy), ∆S° (entropy), and ∆G˚(Gibbs free energy), we used the following Equation^50–52^;

11 $${\text{K}}_{{\text{d}}} =\frac{{{\text{q}}_{{\text{e}}} }}{{{\text{C}}_{{\text{e}}} }}$$

12 $$\Delta {\text{G}}^{{\text{o}}} = - {\text{RT}}\ln \;({\text{K}}_{{\text{d}}} )$$

13 $$\ln {\text{K}}_{{\text{d}}} = {\text{ }}\frac{{\Delta {\text{S}}^{{\text{o}}} }}{{\text{R}}} - \frac{{\Delta {\text{H}}^{{\text{o}}} }}{{{\text{RT}}}}$$

14 $$\Delta {\text{G}}^{{\text{o}}} = \Delta {\text{H}}^{{\text{o}}} - {\text{T}}\Delta {\text{S}}^{{\text{o}}}$$

K_d_ is the distribution coefficient (mL g^−1^), R is the gas constant, and T is the absolute temperature.

### Desorption investigations

The research was done on the desorption of Sr(II) and Eu(III) loaded onto SnMoT sorbent by a batch process with several eluent agents at ambient temperature with a volume-to-sorbent ratio of 100 mL/g. The used eluents are 0.1 M of (HCl, MgCl_2_, CaCl_2_, AlCl_3_, and EDTA). A series of 50 mL bottles, each containing 0.1 g of loaded SnMoT sorbent by Sr(II) and Eu(III) and 10 mL of these eluents was shaken for 24 h, then following the separation of the two phases, the concentrations of Sr(II) and Eu(III) in the solid phase (C_d_) and supernatant (C_s_) were determined in milligrams per liter. The % of desorption was determined using (Eq. 15)^52^:

15 $$\% {\text{Desorption }} = {\text{ }}\left( {\frac{{{\text{C}}_{{\text{S}}} }}{{{\text{C}}_{{\text{d}}} }}} \right)100$$

## Results and discussion

### Adsorbent characterization

#### XRD analysis

**Figure 1:**
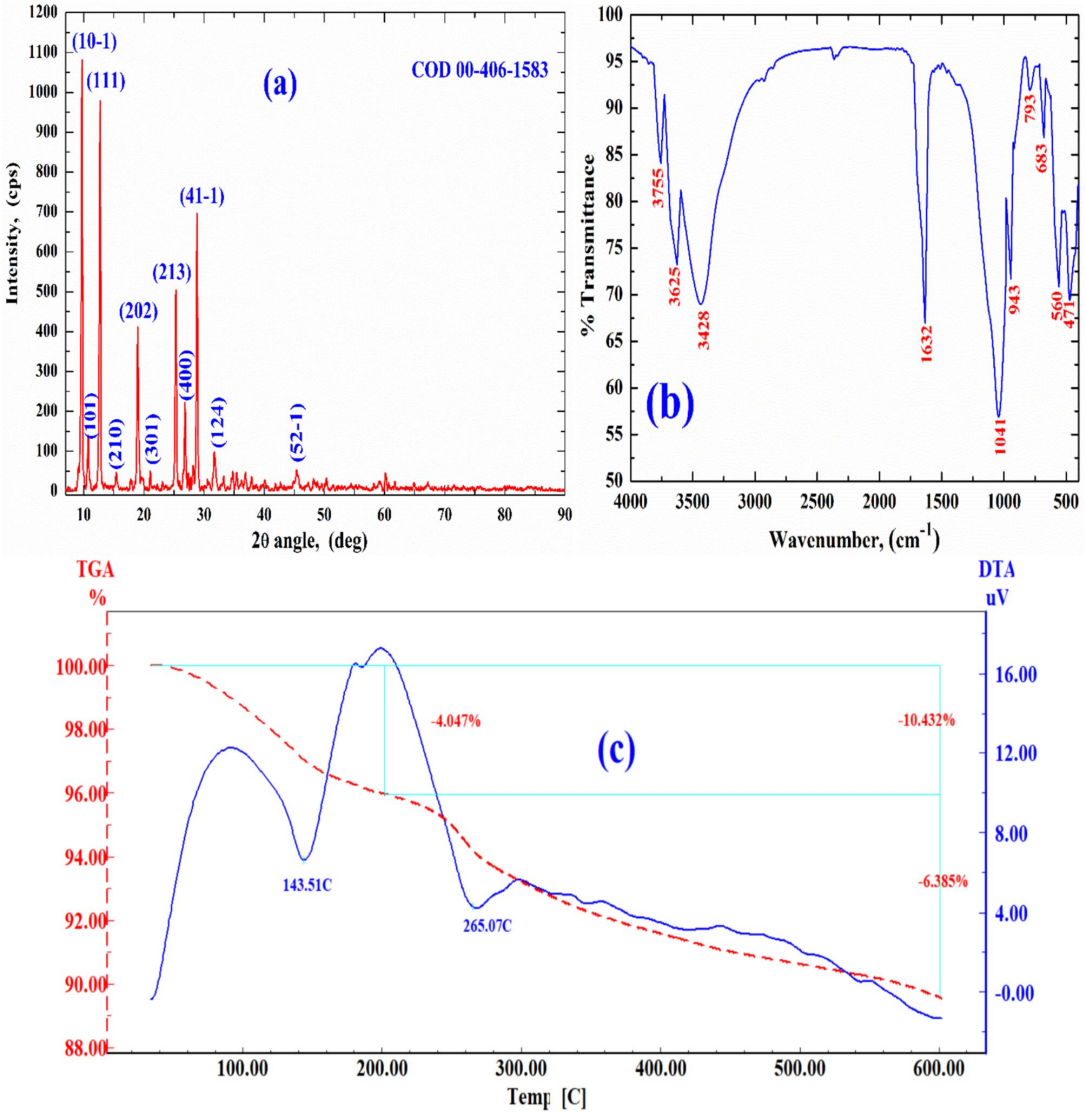
(**a**) XRD analysis (**b**) FT-IR spectrum, and **(c)** TGA and DTA analysis for SnMoT sorbent.

The crystalline character of SnMoT sorbent was examined using X-ray diffraction (XRD), as shown in Fig. 1a. In this Figure, the crystalline structure of SnMoT sorbent is characterized by several sharp peaks at (9.46°, 10.33°, 12.63°, 15.3°, 18.92°, 21.11°, 25.3°, 26.88°, 28.78°, 31.58°, and 45.44°) related to Miller index indications (10-1, 101, 111, 210, 202, 301, 213, 400, 41-1, 124, and 42-5) respectively, with COD 00–406-1583, confirming their crystalline nature with the monoclinic system. This result has the same character as the IPT prepared by Mansy et al.^35^, and P(AA-AN)-talc prepared by Abass et al.^28^.

#### FT-IR analysis

FT-IR spectrum of SnMoT sorbent in Fig. 1b exhibits that the metal-O and metal-OH bands are observed at 560 and 683 cm^−1^ in SnMoT sorbent^53^. Two bands found at 3428 and 1632 cm^−1^ can be explained by intra-structure water molecules' OH frequencies vibrating in a stretched and bowed manner^37^ or attributed to Sn–OH groups^54,55^. Three bands observed at (1041, 793, and 471 cm^−1^) correspond to Sn–O^56^ or due to Si–O, Si–O–Al, and Si–O–Mg bending, respectively^57^. The Sn–O bond vibrations in the Sn–O–Mo matrix are related to FT-IR bands ranging from 700 to 450 cm^−1^ ^55^. The bands at 3625 and 943 cm^−1^ are due to A1–A1–OH (stretching and bending vibration, respectively)^57,58^. The band at 3755 cm^−1^ is related to Al–OH–Mg bonds in talc powder^59^.

#### Thermal analysis

Thermogravimetric analyses (TGA) of SnMoT sorbent (Fig. 1c), revealed a two-stage process when heated at ten °C/min. The 1st stage (32–201 °C) can be related to the desorption of physically adsorbed water from the surface of the sorbent^41,58^. The weight loss in this region is 4.05%. The 2nd stage (201–700 °C) may be due to the loss of chemically bonded H_2_O^41^, the weight loss in this region is 63.39%. Differential thermal (DTA) shows two endothermic peaks at 143 and 265 °C due to free H_2_O and chemically bonded H_2_O loss. From the TGA data in Fig. 1c, the weight loss for SnMoT sorbent continued up to 700 °C. The weight loss of SnMoT sorbent with a heating temperature of 10.43% reflects that SnMoT sorbent is more thermally stable than other sorbents^28^.

#### SEM analysis

**Figure 2:**
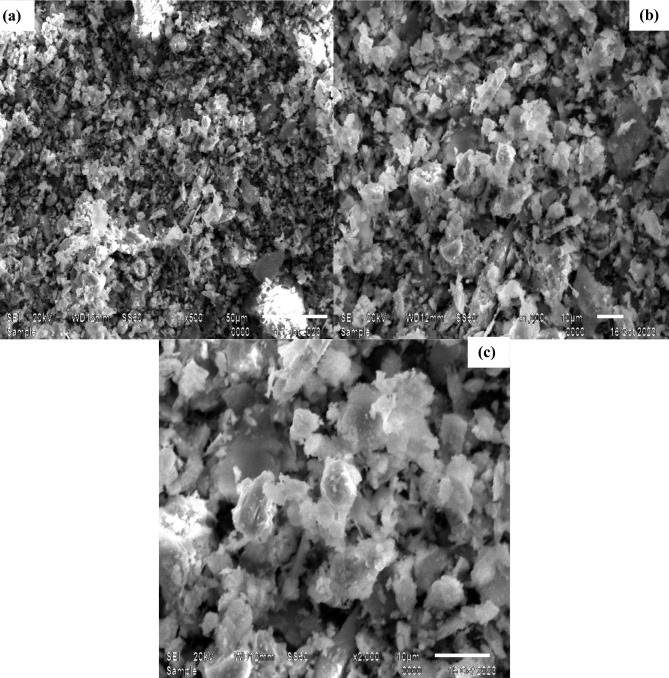
SEM images of SnMoT sorbent at different magnification powers (**a**) X500 (**b**) X1000 (**c**) X2000.

Figure 2 displays SEM pictures of the SnMoT sorbent material at various magnification levels of X500, X1000, and X2000. The findings reveal a varied distribution of tin particles (white) over the molybdate medium (grey); they resemble many tiny islands on the ocean's surface. When the magnification power is increased to X1000 and X2000, the surface appears to have very small pores. These particles are sharp and rough, with intermolecular distances that facilitate the physical sorption process on the substance.

#### XRF analysis

Table 2 contains the elemental analysis of SnMoT sorbent, which can be determined with XRF. These numbers demonstrated that the percentage of metal oxides in the SnMoT sorbent was 34.63, 18.82, 16.93, 16.61, 7.6, and 5.41 for SiO_2_, MgO, MoO_3_, SnO_2_, Fe_2_O_3_, and Al_2_O_3_, respectively. These results verified that every component found in SnMoT sorbent is present.

**Table 2:** Elemental analysis of SnMoT sorbent using X-ray fluorescence.

Component	SiO_2_	MgO	MoO_3_	SnO_2_	Fe_2_O_3_	Al_2_O_3_
Concentration (%)	34.63	18.82	16.93	16.61	7.60	5.41

### Chemical stability

Table 3 shows the solubility test of SnMoT sorbent toward various solvents, which reflects that the SnMoT sorbent was very steady in common mineral acids and alkalies. These data are useful for the sorption process in different media. Table 3 demonstrates that, in comparison to other sorbents, SnMo sorbent has comparatively high chemical stability^60–62^.

**Table 3:** Chemical stability of SnMoT sorbent in different solvents.

Solvents	% Solubility
DDW	Below detection limit
0.5 mol/L HNO_3_	1.02
1 mol/L HNO_3_	1.92
2 mol/L HNO_3_	2.36
4 mol/L HNO_3_	6.75
0.5 mol/L HCl	1.53
3 mol/L HCl	2.6
0.5 mol/L HCl	3.82
1 mol/L HCl	7.65
2 mol/L NaOH	3.2
4 mol/L NaOH	18.3

### Metal hydrolysis process

The side reaction of metal hydrolysis, which mostly depends on the pH of the solution, primarily affects the separation of the examined elements by the suggested adsorbent. In this context, several tests have been conducted separately. As a result, samples (10 mL each) with 50 mg/L of each element (in distilled water) were made independently at various pH levels ranging from 2 to 9. Samples were shaken for 30 min before being filtered, and every element's concentration at every pH level was tested spectrophotometrically to calculate the precipitation %, Table 4. Sr(II) and Eu(III) precipitated after pH 8 and 5, respectively. The results of metal hydrolysis show that all subsequent studies were conducted at pH 6 and 4 for Sr(II) and Eu(III), respectively, to avoid the hydrolysis of the metal ions.

**Table 4:** Sr(II) and Eu(III) hydrolysis (%) at different pHs (1–9) [C_o_ = 50 mg/L and time = 0.5 h].

pH	% Hydrolysis	
	Sr(II)	Eu(III)
2.0	Nil	Nil
3.0	Nil	Nil
4.0	Nil	Nil
5.0	Nil	7.5
6.0	Nil	49.8
7.0	Nil	100
8.0	93.5	100
9.0	61.8	100

### Study of sorption

The batch method was used to sorb Sr(II) and Eu(III) from aqueous solutions using the SnMoT sorbent. The different parameters influencing the individual studies of Sr(II) and Eu(III) sorption optimize their sorption on the synthesized SnMoT sorbent. The following sections detail the results that were achieved.

### Effect of pH

**Figure 3:**
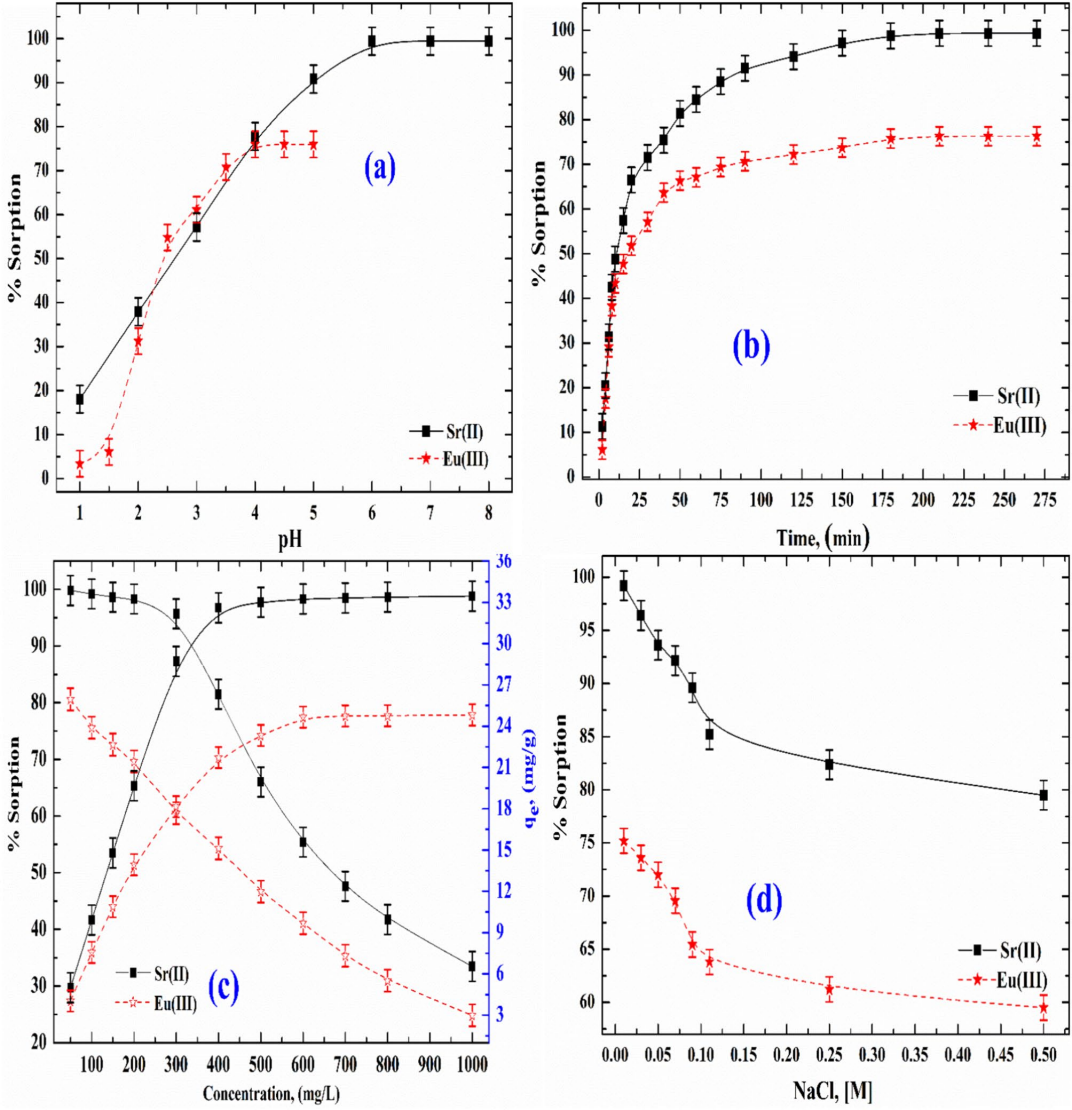
Sorption of Sr(II) and Eu(III) onto SnMoT sorbent (**a**) Effect of pH on the % sorption, (**b**) Effect of shaking time on the % sorption, (**c**) Effect of initial concentration on the % sorption, and (**d**) Effect of ionic strength on the % sorption.

The % sorption of Sr(II) and Eu(III) from aqueous solutions by the SnMoT sorbent was studied with initial concentration (C_o_) 100 mg/L, batch factor (V/m) = 100 mL/g, shaking time (24 h), and pH = (1–8) for Sr(II) and pH = (1–5) for Eu(III) as shown in Fig. 3a. From this Figure, the % sorption increases with increasing pH (1–6) from 18.0 to 99.4% for Sr(II) and at pH (1–4) from 3.4 to 75.9% for Eu(III). Above this pH value, no change was observed for the % sorption, and all experimental work was done at pH 6 and 4 for Sr(II) and Eu(III), respectively. Additionally, it was noted that the percentage of Sr(II) and Eu(III) sorption is low at low pH levels. This is most likely because the surface active sites are protonated, and the amount of H_3_O^+^ ions in the aqueous solution increases. Consequently, the competition for the accessible binding surface active site between H_3_O^+^ and Sr(II) and Eu(III) was brought about by the positively charged surface sites that decreased Sr(II) and Eu(III) uptake. The concentration of OH^−^ ions grew, and the concentration of H_3_O^+^ ions decreased as the original pH values increased, resulting in surface deprotonation of sorbents; these findings indicate that the SnMoT sorbent's surface typically has a negative charge. As a result, there was more attraction between the sorbent's surface and the solution's positive charge of metal ions.

### Influence of shaking time

The effect of contact time on % sorption of Sr(II) and Eu(III) onto the synthesized SnMoT sorbent was studied, initial concentration (C_o_) = 100 mg/L, batch factor (V/m) = 100 mL/g, shaking time (2–270 min), pH = 6 and 4 for Sr(II) and Eu(III), respectively. The obtained data are represented in Fig. 3b and show that the % sorption of Sr(II) and Eu(III) onto the synthesized SnMoT increased over time, reaching equilibrium at about 210 min. The rate of Sr(II) and Eu(III) sorption onto SnMoT sorbent rapidly increases from 2 to 180 min and slowly increases from 180 to 210 min, after which there is no change in the uptake, for additional experimental work, 210 min was utilized as the equilibrium time.

### Influence of concentration

Figure 3c reveals the plots between % sorption and amount uptake q_e_, (mg/g) of Sr(II) and Eu(III) onto SnMoT sorbent and C_o_ at the range (50–1000 mg/L) at a fixed temperature (298 ± 1 K), batch factor (V/m) = 100 mL/g, shaking time (210 min), pH = 6 and 4 for Sr(II) and Eu(III), respectively. The % sorption of Sr(II) and Eu(III) onto SnMoT sorbent decreases as the initial concentration of Sr(II) and Eu(III) increases. These data reflect that the % sorption is very high at a small initial concentration due to low competition. Also, the data represented in Fig. 3c reflect that q_e_ of Sr(II) and Eu(III) increases as the initial concentration of Sr(II) and Eu(III) increases and the maximum q_e_ (33.45 and 24.82 mg/g for Sr(II) and Eu(III), respectively) carried out at initial concentration 1000 mg/L.

### Influence of ionic strength

Plots of the ionic strength of NaCl (0.01–0.5 M) and the percentage of sorption of Sr(II) and Eu(III) onto SnMoT sorbent are displayed in Fig. 3d. The experiment was carried out at [C_o_ = 100 mg/L, V/m = 100 mL/g, agitating time 210 min, pH = 6 and 4 for Sr(II) and Eu(III), respectively]. As ionic strength increases, Fig. 3d shows a modest decrease in the percentage of sorption of Sr(II) and Eu(III), leading to ionic strength independence. The independence of strong ionic strength is mainly dominated by inner-sphere surface complexation^63^.

### Kinetic study

**Figure 4:**
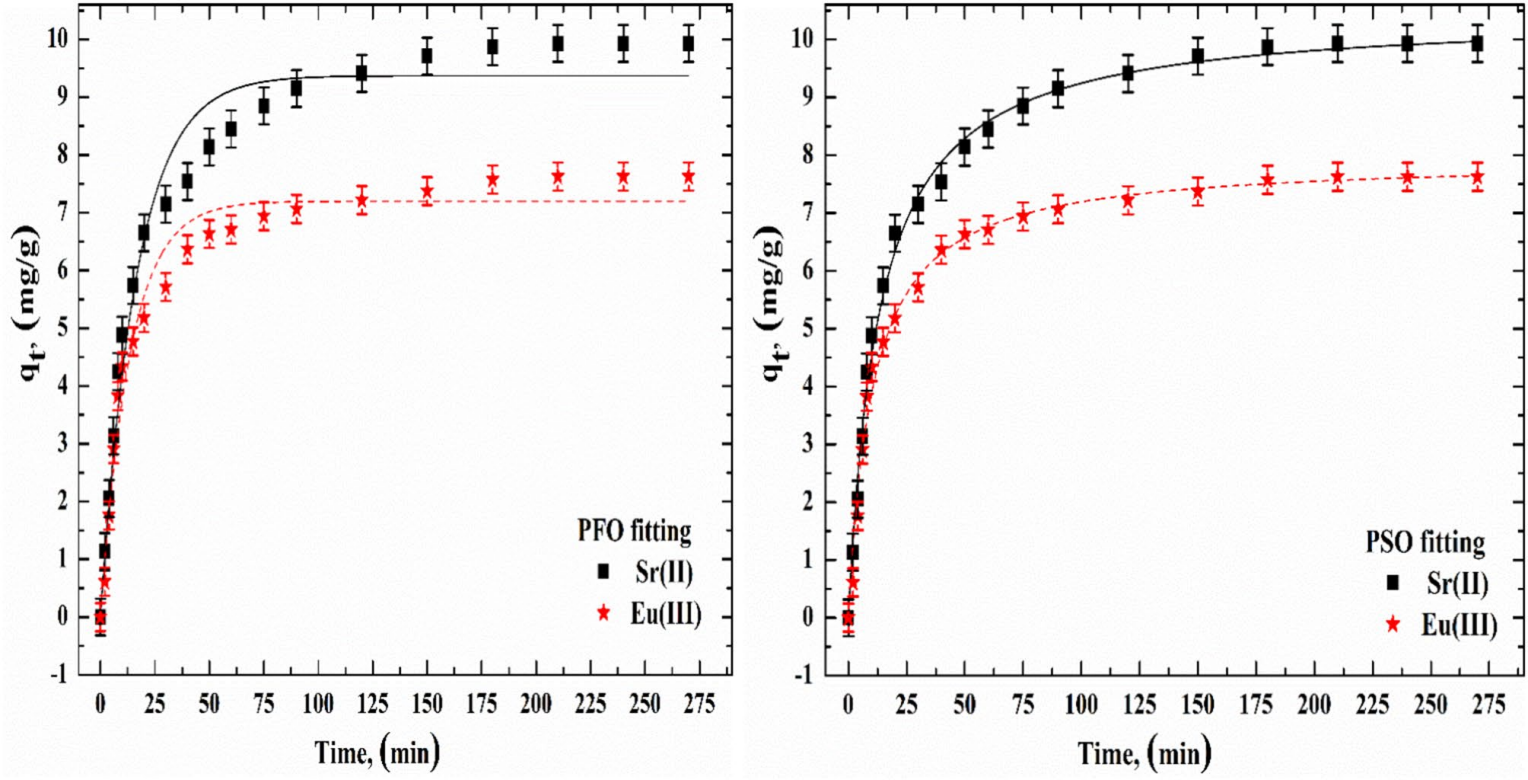
Kinetic modelling fitting of Sr(II) and Eu(III) onto SnMoT sorbent at [C_o_ = 100 mg/L, V/m = 100 mL/g, pH = 6 and 4 for Sr(II) and Eu(III), respectively].

The adsorption kinetics were examined by applying the PFO and PSO model equations to the experimental data. Two steps are involved in the adsorption of Sr(II) and Eu(III) onto SnMoT sorbent (Fig. 4). For 180 min, the first step involved rapid adsorption. In the second stage, adsorption was slower and longer, presumably affecting the interior of the adsorbent. The initial phase was swift and dominated in terms of numbers; the second, however, was less rapid and had no quantitative impact. During the first adsorption phase, the SnMoT surface had several accessible active centers. Following the occupation of these centers, the equilibrium condition was attained, and the second stage, which included the interior regions of the adsorbent, was started. The high concentration of active centers on the surface of SnMoT sorbent causes the fast stage; however, during the slower stage, the adsorption process's effectiveness is decreased as these sites fill more fully. During the initial adsorption stage, several active centers are on the SnMoT sorbent surface. These active centers are adsorbed with Sr(II) and Eu(III). As time passes, the number of active centers on the SnMoT sorbent surface grow saturated with Sr(II) and Eu(III), then Sr(II) and Eu(III) gradually diffuse through the SnMoT sorbent's pore in the following step. When the PFO and PSO models' R^2^ values (Table 5) were contrasted, it was found that the PSO model fit the data better regarding kinetics. Additionally, the proximity of compatibility between the experimental and theoretically derived qe values was demonstrated with the PSO model. These findings showed that the adsorption process followed the PSO rate kinetics. Additionally, the Chi-square (χ^2^) is considerably used to determine the differences between values concluded by a model and the values observed experimentally as it has the lowest value of χ^2^. As shown in Table 5, the χ^2^ of the PSO model was lower than that of the PFO model, indicating the applicability of the PSO.

**Table 5:** Kinetic parameters and correlation coefficients (R^2^) for the sorption of Sr(II) and Eu(III) onto SnMoT sorbent.

Metal ions	PFO		q_e_ (exp.)	PSO	
Sr(II)	q_e_(cal.)	9.37	9.940	q_e_(cal.)	10.45
	K_1_	0.060		K_2_	0.007
	H_1_	0.562		H_2_	0.80
	R^2^	0.973		R^2^	0.996
	χ^2^	0.034		χ^2^	0.025
Eu(III)	q_e_(cal.)	7.20		q_e_(cal.)	7.94
	K_1_	0.074		K_2_	0.012
	H_1_	0.530		H_2_	0.76
	R^2^	0.972		R^2^	0.990
	χ^2^	0.026		χ^2^	0.0122

#### Sorption isotherms

**Figure 5:**
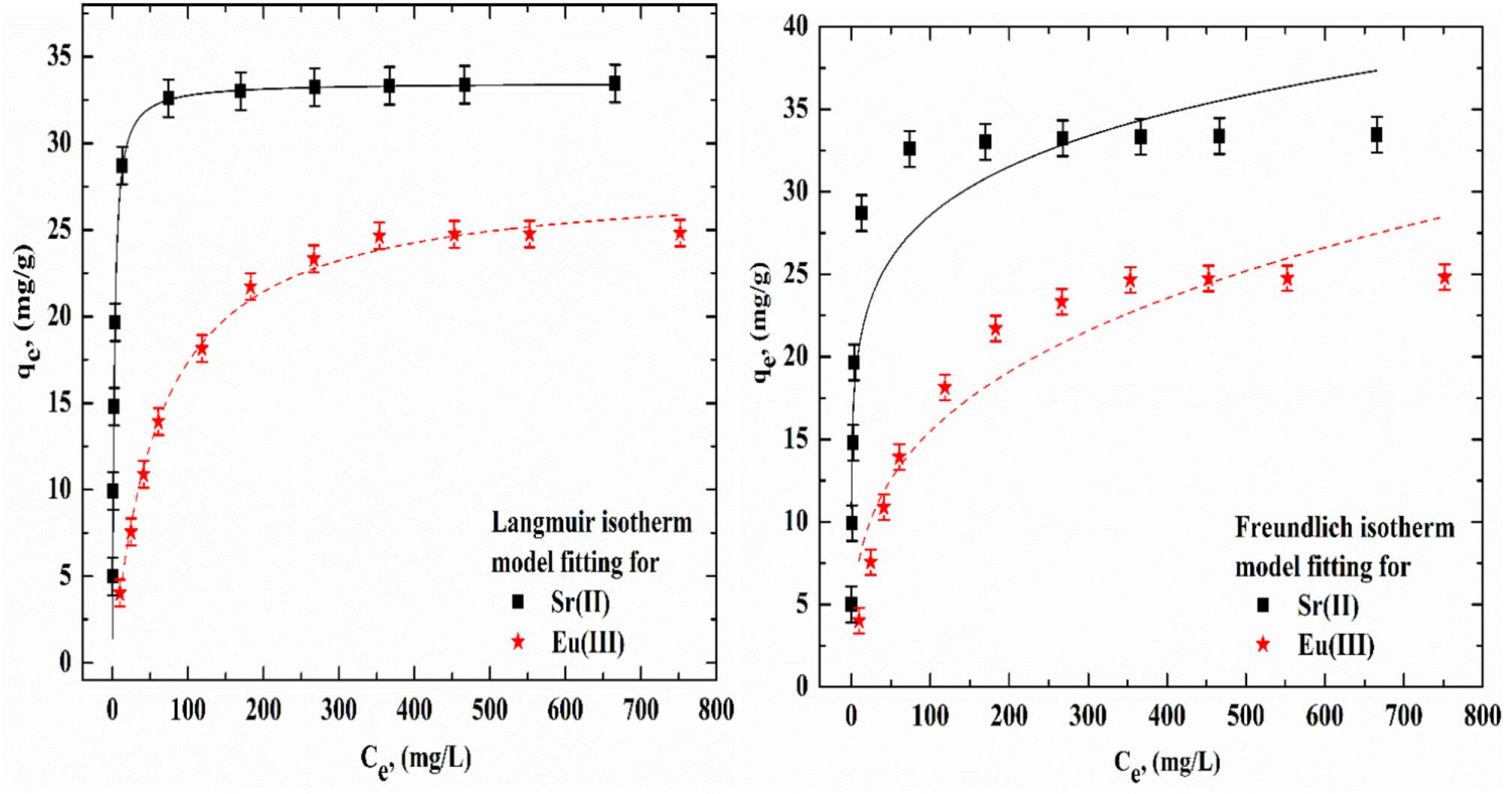
Isothermal modeling fitting of Sr(II) and Eu(III) onto SnMoT sorbent at [Eq. time = 210 min, V/m = 100 mL/g, pH = 6 and 4 for Sr(II) and Eu(III), respectively].

Various isotherm models were employed to examine the equilibrium data and determine a suitable model for the design procedure. The Langmuir and Freundlich isotherm equations examined Sr(II) and Eu(III) sorption onto SnMoT sorbent. The correlation coefficients (R^2^) consistently demonstrate the applicability of isotherm equations. The interaction mechanism between SnMoT and Sr(II) and Eu(III) at equilibrium was determined using adsorption isotherms. When the R^2^ values from the Langmuir and Freundlich isotherm models are compared (Fig. 5, and Table 6), the adsorption process of Sr(II) and Eu(III) followed the Langmuir isotherm which offered a better fit with R^2^ = 0.985 and 0.994 for Sr(II) and Eu(III), respectively. The results of R_L_ values were (0.0023 and 0.057), reflecting the favorable sorption isotherms of Sr(II) and Eu(III)^64^. The highest amount of sorption that could be achieved was 33.5 and 28.0 mg/g for Sr(II) and Eu(III), respectively. However, the use of R^2^ is limited to solving non-linear forms of isotherm equations, but not the errors in isotherm curves. In this concern, it is necessary to analyze the data set using the chi-square test statistic to assess the best-fit isotherm for the sorption system Eq. (10). According to the data in Table 6, by comparing the values of χ^2^ for different isotherms, it was found that the lower χ^2^ values of Langmuir model pointed to the best fitting isotherm for the sorption of Sr(II) and Eu(III) onto SnMoT sorbent. Therefore, sorption isotherm data are better simulated by the Langmuir model rather than the Freundlich model. This reveals that monolayer sorption was the main interaction mechanism of Sr(II) and Eu(III) with SnMoT sorbent used. These findings verified that the Langmuir model is more applicable for the adsorption of Sr(II) and Eu(III) onto SnMoT sorbent.

**Table 6:** Isotherm parameters for sorption of Sr(II) and Eu(III) onto SnMoT sorbent.

Metal ions	qe(Exp.)	Langmuir				Freundlich			
		q_m_	R_L_	R^2^	χ^2^	1/n	K_F_	R^2^	χ^2^
Sr(II)	33.45	33.5	0.0023	0.985	9.1E-05	7.094	14.93	0.842	18.5
Eu(III)	24.82	28.0	0.057	0.994	3.5E-01	3.305	3.84	0.889	21.0

#### Thermodynamic studies

**Figure 6:**
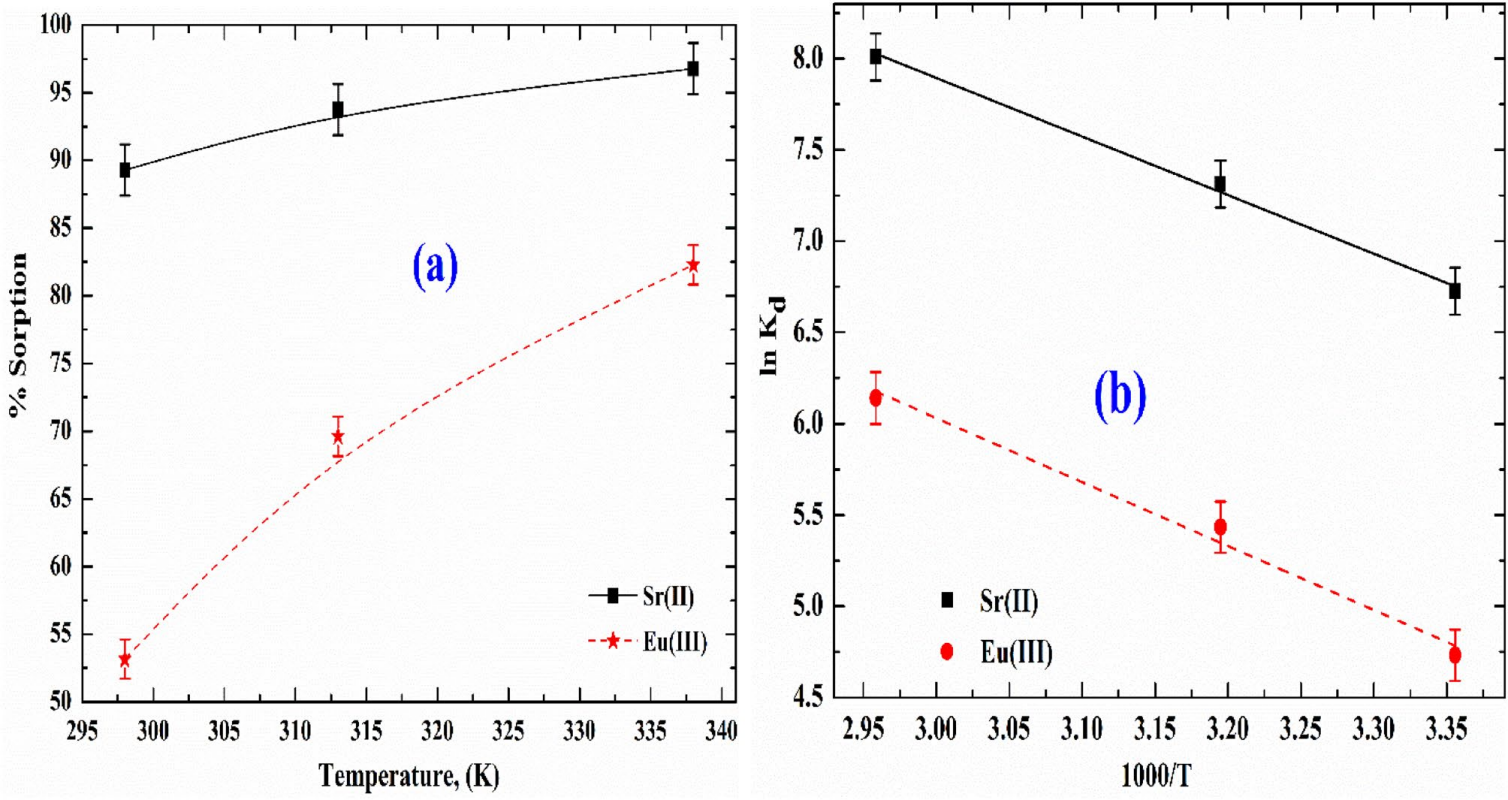
(**a**) Effect of reaction temperature on the % sorption of Sr(II) and Eu(III) onto SnMoT sorbent and (**b**) Van’t Hoff plot of the adsorption of Sr(II) and Eu(III) onto SnMoT sorbent.

The influence of temperature on the % sorption of Sr(II) and Eu(III) by SnMoT sorbent was studied at an initial concentration of 200 mg/L, pH = 6 and 4 for Sr(II) and Eu(III), respectively, and shaking time = 210 min and the result is represented in Fig. 6a. This Figure illustrates how the endothermic nature of the sorption process is reflected by an increase in the % sorption of Sr(II) and Eu(III) with increasing reaction temperature. Temperatures of 298, 313, and 338 K were investigated to interpret the thermodynamic behavior of the adsorption process (Fig. 6b). The change in ∆H° during the adsorption process was 26.7 and 29.1 kJ/mol for Sr(II) and Eu(III), respectively. Temperature increase showed a positive influence on Sr(II) and Eu(III) elimination in the endothermic adsorption process. With the rising temperature, the amount adsorbed increased. The entropy change, ΔS°, was 145.8 and 137.6 J/mol.K for Sr(II) and Eu(III), respectively. This finding revealed that the adsorption process was random. A positive entropy could be regarded as an increase in the randomness of the adsorption system as a result of the adsorbent's high affinity^65^. ΔG°s were − 16.7, − 18.9, and − 22.6 kJ/mol for Sr(II), also ΔG°s were − 11.9, − 13.9, and − 17.4 kJ/mol for Eu(III). The more extensive availability of ΔG˚ at higher temperatures was related to increased mobility of Sr(II) and Eu(III) sorbed onto the SnMoT sorbent surface, increased electrostatic interaction among metal ions sorbed and different active groups on the SnMoT sorbent surface.

#### Desorption investigations

Sr(II) and Eu(III) loaded onto SnMoT sorbent were desorbed using a variety of desorbing agents, and Table 7 displays the data. The results show that washing with AlCl_3_ hardly desorbed the Sr(II) from the adsorbent surface. While it is relatively desorbed by washing with MgCl_2_, CaCl_2_, and EDTA solutions and using 0.1 M HCl as the eluent, high desorption of Sr(II) loaded onto SnMoT sorbent was achieved (96.8%). However, Table 7 illustrates that the Eu(III) was hardly desorbed from the adsorbent surface by washing with MgCl_2_, CaCl_2_, and EDTA. While it is relatively desorbed by washing with AlCl_3_ great desorption of Eu(III) loaded onto SnMoT sorbent was reached using 0.1 M HCl as eluent (92.9%). As a result, the order of the eluents' efficiency in releasing Sr(II) from the loaded SnMoT sorbent: HCl (96.8%) >  > EDTA (51.6%) > CaCl_2_ (38.5%) > MgCl_2_ (34.6%) > AlCl_3_ (8.5%). The order is followed by the effectiveness of the eluents employed to release Eu(III) from the loaded SnMoT sorbent: HCl (92.9%) >  > AlCl_3_ (39.6%) > EDTA (29.5%) > MgCl_2_ (22.5%) > CaCl_2_ (18.6%). According to the results, it is possible to effectively recover Sr(II) and Eu(III) from loaded SnMoT sorbent with intriguing yields using 0.1 M HCl.

**Table 7:** Desorption of Sr(II) and Eu(III) loaded onto SnMoT sorbent using different eluents.

Desorbing agents, 0.1 M	% Desorption	
	Sr(II)	Eu(III)
HCl	96.8	92.9
MgCl_2_	34.6	22.5
CaCl_2_	38.5	18.6
AlCl_3_	8.5	39.6
EDTA	51.6	29.5

## Conclusion

The SnMoT sorbent was prepared using the precipitation process. The SnMo sorbent was described and used to sorb Sr(II) and Eu(III) in batch technique from aqueous solutions. The produced sorbent's equilibrium time (210 min) is confirmed by the sorption data of Sr(II) and Eu(III), obeys the kinetic model of pseudo-2nd order, and is more fitting for the Langmuir isotherm with the highest possible sorption capacity was 33.5 and 28.0 mg/g for Sr(II) and Eu(III), respectively. The thermodynamic parameters displayed that the sorption process was spontaneous and endothermic, suggesting favorable adsorption under the tested conditions. 0.1 M HCl show optimum desorption of Sr(II) and Eu(III). Finally, the obtained results reveal the applicability of the fabricated sorbent as a substance that effectively absorbs Sr(II) and Eu(III) from aqueous solutions and can be used as a promising sorbent.

## Data Availability

All data generated or analyzed during this study are included in this published article. Datasets are available in the manuscript.
